# Management of autosomal dominant hypocalcemia type 1: Literature review and clinical practice recommendations

**DOI:** 10.1007/s40618-024-02496-y

**Published:** 2024-11-28

**Authors:** Thomas De Coster, Karel David, Jeroen Breckpot, Brigitte Decallonne

**Affiliations:** 1 General Internal Medicine, University Hospitals Leuven, Leuven, Belgium; 2 Endocrinology, University Hospitals Leuven, Leuven, Herestraat 49, 3000 Belgium; 3 Center for Human Genetics, University Hospitals Leuven, Leuven, Belgium

**Keywords:** Autosomal Dominant Hypocalcemia type 1, Calcium sensing receptor, Parathyroid hormone analogues, Calcilytics, Practice recommendations

## Abstract

**Purpose:**

Autosomal Dominant Hypocalcemia type 1 (ADH1), caused by gain-of-function variants in the calcium-sensing receptor (CASR), is characterized by a variable degree of hypocalcemia and hypercalciuria with inappropriately low PTH. The clinical spectrum is broad, ranging from being asymptomatic to presenting with severe clinical features of hypocalcemia and end-organ damage such as nephrolithiasis and intracerebral calcifications. Although the underlying pathophysiology is different, ADH1 patients are often managed as patients with ‘classical’ primary hypoparathyroidism, possibly leading to (exacerbation of) hypercalciuria. New treatments such as PTH analogues and calcilytics directly targeting the CASR are in the pipeline. Specific clinical guidance for treatment and monitoring of ADH1 patients is lacking. The purpose of this study is to provide a literature review on management of ADH1, including new therapies, and to formulate practice recommendations.

**Methods:**

We searched for articles and ongoing clinical trials regarding management of ADH1.

**Results:**

Forty articles were included. First we review the conventional treatment of ADH1, focusing on active vitamin D, calcium supplements, thiazide diuretics, phosphorus binders and dietary recommendations. In a second part we give an overview of studies with emerging treatments in ADH1: PTH analogues (PTH1-34, rhPTH1-84, TransCon PTH and others) and calcilytics (preclinical studies and clinical trials). In a third part we discuss literature findings regarding monitoring of ADH1 patients. Finally, we formulate clinical practice recommendations.

**Conclusion:**

We provide an overview of conventional and new treatments for ADH1 patients. Based on these data, we propose practical recommendations to assist clinicians in the management of ADH1 patients.

**Supplementary Information:**

The online version contains supplementary material available at 10.1007/s40618-024-02496-y.

## Introduction

Autosomal Dominant Hypocalcemia type 1 (ADH1) (OMIM 601198), also known as Familial Hypocalcemic Hypercalciuria, is the most common genetic form of isolated hypoparathyroidism (hypoPT) with an estimated prevalence of 3.9 per 100 000 [1]. ADH1 is caused by gain-of-function variants in the *CASR* gene, encoding the calcium-sensing receptor (CASR), the main receptor involved in the regulation of calcium homeostasis [2]. To date, over 100 unique *CASR* gain-of-function variants have been described [3]. CASR is mostly expressed on the surface of the parathyroid chief cells [2]. High extracellular calcium levels stimulate the parathyroid CASR, resulting in an increase in intracellular calcium, inhibition of PTH synthesis and secretion, inhibition of parathyroid cellular proliferation, and promotion of intracellular degradation of PTH [2, 4, 5]. CASR is also highly expressed in the basolateral membrane of the thick ascending loop of Henle (TAL) [6]. Upon activation, the expression of pore-blocking claudin-14 increases, causing reduced permeability of the tight junctions, decreased paracellular calcium reabsorption and thus an increase in urinary calcium excretion [6]. The effects of CASR activation in organs involved in calcium homeostasis are shown in Table 1 [6–10].

**Table 1:** The role of CASR in calcium homeostasis

Organ	Mechanism	Effect on calcium homeostasis
Parathyroid glands	↓ PTH production and secretion ↓ Parathyroid cellular proliferation ↑ PTH intracellular degradation	↓ Renal 1,25(OH)_2_D_3_ synthesis ➔↓ intestinal Ca^2+^absorption ↓ Renal Ca^2+^reabsorption ↓ Bone resorption
Kidney	↑ expression of claudin-14 in TAL ➔ ↓ permeability of tight junctions	↓ Renal Ca^2+^reabsorption
Bone	↓ Osteoclast activation ↑ Osteoblast recruitment, proliferation and differentiation	↓ Bone turnover
Intestine	↓ TRPV6 expression	↓ Intestinal Ca^2+^absorption
Thyroid	↑ Calcitonin secretion	↓ Bone resorption
Breast	↓ PTHrP production ↑ PMCA2 activity	↓ Bone resorption ↑ Ca^2+^ transport from the mammary cells into the milk

Abbreviations: 1,25(OH)2D3: calcitriol; PMCA2; plasma membrane Ca^2+^-ATPase 2; PTH: parathyroid hormone; PTHrP; PTH-related peptide; TAL: thick ascending limb; TRPV6: transient receptor potential vanilloid 6

Activating *CASR* variants increase the sensitivity of the CASR to extracellular ionized calcium (Ca^2+^ _e_) [2, 5, 7, 11]. This results in an abnormal suppression of PTH synthesis and secretion at physiological levels of Ca^2+^ _e_ leading to hypocalcemia and relative hypercalciuria [2, 4–6]. Activating *CASR* variants have independent effects in the kidneys, leading to an even more pronounced urinary calcium excretion for any given serum calcium level. As such, hypercalciuria in ADH1 is a result of both aforementioned mechanisms [2].

ADH1 patients are biochemically characterized by hypocalcemia with inappropriately low but detectable or normal PTH levels, high normal or elevated phosphate, and normal or low levels of magnesium [2, 12, 13]. About 50% of patients have hypercalciuria. Hypercalciuria is usually defined as > 6.25 mmol/24 h urine (females) and > 7.5 mmol/24 h urine (males), or as a urinary calcium-to-creatinine ratio > 0.6 if no (reliable) 24 h urine collection is available [6, 14]. There is no specific definition of hypercalciuria in ADH1 patients. A subgroup of *CASR* gain-of-function variants leads to renal loss of sodium, chloride and magnesium resulting in hyperreninemia, hyperaldosteronism with normal blood pressure, hypokalemia and metabolic alkalosis, in addition to hypocalcemia and relative hypercalciuria. This combination of biochemical features is referred to as “ADH with Bartter-like syndrome”, and is caused by an increased CASR activity that inhibits the Na-K-2Cl cotransporter leading to inhibition of NaCl transport in the TAL [15–17].

Acute symptoms of ADH1 are mainly caused by hypocalcemia [13]. Hypocalcemia depolarizes the resting membrane potential in neurons, thereby increasing the probability of triggering action potentials. This results in neuromuscular irritability, which is the signature symptom of hypocalcemia. Sensory neuronal irritability manifests as paresthesia in the extremities and the peri-oral and oral area. Motor neuronal irritability manifests as muscle spasms or tetany, ranging from carpopedal spasms to life-threatening laryngospasms [18]. The severity of these neurological symptoms is inversely related to serum calcium levels [19]. Seizures have been described in 39% of ADH1 patients and particularly occur in younger patients, often during febrile episodes [3, 13, 18–21]. Long-term complications of chronic hypocalcemia and hyperphosphatemia include central nervous system calcifications, nephrocalcinosis, kidney stones, impaired renal function, cognitive dysfunction and posterior subcapsular cataract [4, 18, 22–25]. Ectopic central nervous system calcifications occur in 32% of ADH1 patients and are mostly observed in the basal ganglia [3, 5, 9, 10]. They can also appear in the grey and white matter junction, thalamus, dentate nucleus and cerebellar parenchyma [18]. Recently, Zavatta et al. found that basal ganglia calcifications in chronic hypoPT patients are correlated with low serum calcium levels and low calcium/phosphate ratio but not with phosphate or the calcium-phosphate product [26]. The clinical relevance of these calcifications remains unclear [27]. An association with extrapyramidal features such as Parkinsonism has been proposed, however causality has not yet been established [28]. Nephrocalcinosis occurs in 36% of ADH1 patients [13, 18]. Abnormal dentition development with hypoplastic enamel, shortened roots and hypoplastic or absent teeth have also been described [29]. In addition, myopathy of the skeletal muscles, associated with increased levels of creatine kinase and a reduction in muscle strength, can be a sign of ADH1. A dry scaly skin, brittle nails and onycholysis are also commonly observed [18]. More rare symptoms of ADH1 include cardiac arrythmias, hypocalcemia-associated dilated cardiomyopathy and heart failure [18, 30]. About one-third (27%) of ADH1 patients remain asymptomatic. These patients have a significantly higher serum calcium concentration than symptomatic ADH1 patients [13]. However, asymptomatic patients can still exert complications, such as nephrocalcinosis and basal ganglia calcifications [27].

Conventional treatment of ADH1 consists of active vitamin D analogues and/or calcium supplements, aiming at alleviating symptoms [3]. Furthermore, thiazides and a low sodium diet may be considered. PTH analogues and calcilytics represent emerging treatments. Raising serum calcium levels does however not always decrease symptoms and may induce or exacerbate pre-existing hypercalciuria, increasing the risk for long-term renal complications [5, 31]. It is unclear whether asymptomatic patients need any treatment, and if so which treatment modality is to be preferred. Although ADH1 represents a separate disease entity, ADH1 patients are often managed similarly to patients with postoperative primary hypoPT.

The purpose of this study is to provide a literature review focusing on management of ADH1, including emerging therapies, and to formulate clinical practice recommendations regarding treatment and monitoring for the clinician following ADH1 patients.

## Methods

**Fig. 1:**
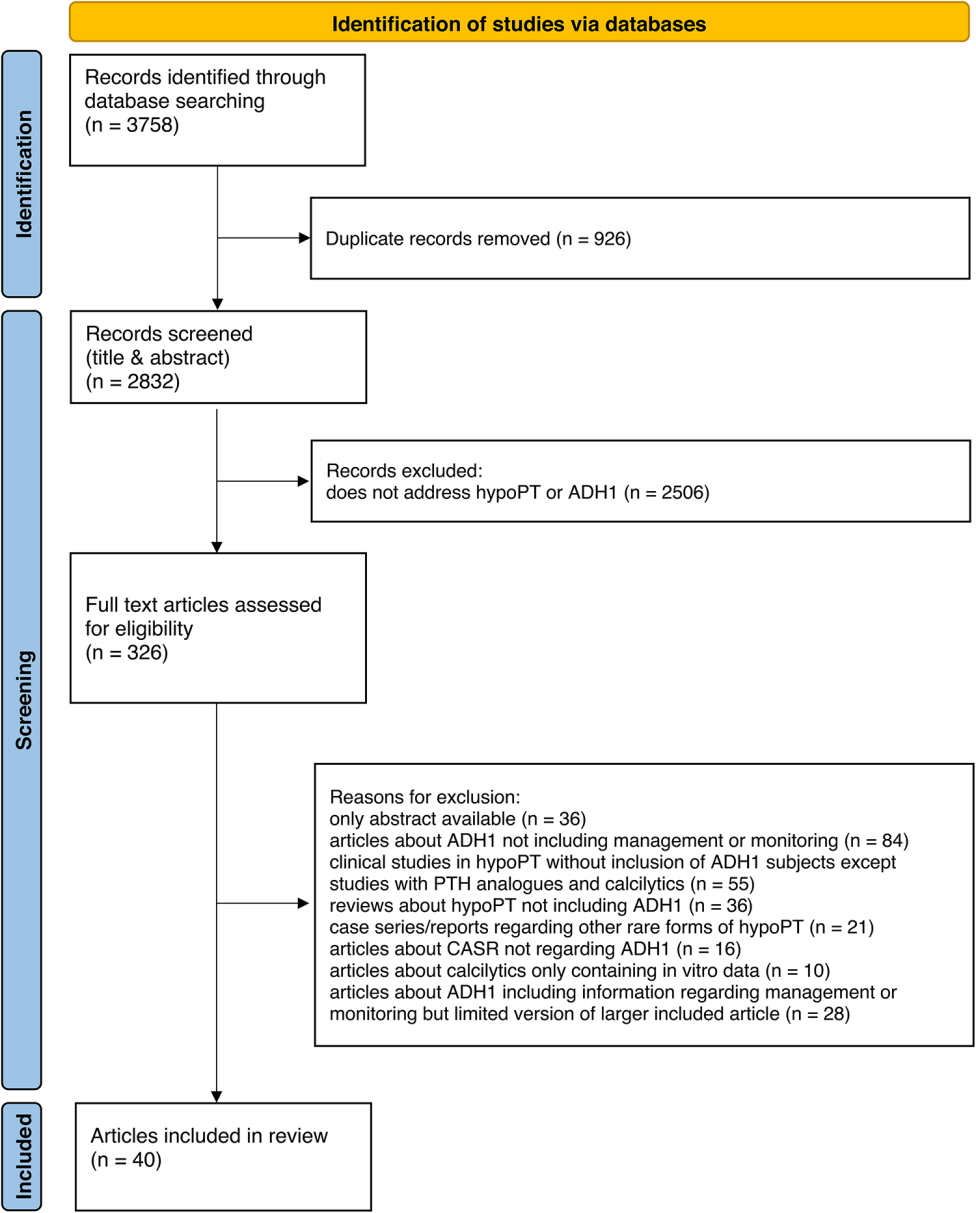
PRISMA Flow diagram of the study selection process. ADH1: Autosomal Dominant Hypocalcemia type 1; CASR: calcium sensing receptor; hypoPT: hypoparathyroidism; QoL: quality of life

We performed a review about the management of ADH1 patients and provided clinical practice recommendations. For this, we searched for articles regarding management of ADH1 in the following databases: Pubmed, Embase, Cochrane Library and Trip database. We used modified search strategies for each database. Furthermore, we searched through controlled-trials.com and clinicaltrials.gov for ongoing clinical trials. We also searched for grey literature in MedRxiv and BioRxiv. Details of the search strategy can be found in Supplemental Data. The search was updated until November 12, 2023. The identification and screening process is described in detail in Fig. 1.

## Results

### Study selection

We included 40 articles including 15 clinical trials, 1 retrospective cohort study, 1 survey, 3 case reports/series, 1 systematic review, 8 narrative reviews, 4 guidelines and 7 animal studies (Fig. 1).

### Conventional treatment of ADH1

#### Vitamin D analogues and calcium supplementation

Roszko et al. reported that in a cohort of 57 ADH1 patients, 59% were prescribed only active vitamin D analogues, 2% only calcium supplementation, and 39% a combination of both. In addition, 14% of ADH1 patients also received magnesium supplementation [3].

Since PTH levels are low or even undetectable in ADH1 patients, PTH-dependent renal 1-alpha-hydroxylation of 25(OH)D_3_ is impaired [19]. Therefore, calcitriol (1,25(OH)_2_D_3_) or alfacalcidol (1α(OH)D_3_) are the vitamin D analogues of choice in the management of ADH1 [18, 19]. In the treatment of hypoPT, these vitamin D analogues are titrated to achieve serum calcium levels within or just below the lower normal reference range for calcium [18, 32]. Adults and older children typically receive calcitriol doses between 0.25 and 2 µg/d, while infants and young children usually need 0.01–0.04 µg/kg/d [18, 33]. Low levels of 25(OH)D_3_ can lead to muscle weakness, which can be mistakenly considered a symptom of hypocalcemia, and some tissues can generate 1,25(OH)_2_D_3_ from 25(OH)D_3_ independently of PTH [34, 35]. Therefore, ergocalciferol and cholecalciferol supplementation could still be beneficial to ADH1 patients. In the 2015 European Society of Endocrinology (ESE) guidelines regarding management of hypoPT in adults, a target concentration of 20ng/mL is proposed as adequate [34].

ESE recommends oral calcium supplements at a total daily dose of 800–2000 mg of elemental calcium [34]. Calcium carbonate is most often used, since it is the least expensive and has the highest content of elemental calcium (40%) [18]. For efficient absorption, calcium carbonate requires an acidic environment. Therefore, it should be taken together with a meal [34, 36]. Calcium citrate (20% elemental calcium), calcium gluconate and calcium lactate are possible alternatives [18, 33]. ESE recommends calcium citrate to patients with achlorhydria or treated with proton pump inhibitors (PPIs) [34, 37]. Furthermore, calcium citrate may be useful in patients suffering from constipation under calcium carbonate supplementation [33]. Calcium phosphate is contraindicated considering the risk of hyperphosphatemia [38].

Furthermore, according to guidelines by the First International Conference on the Management of Hypoparathyroidism and the Second International Workshop, IV calcium gluconate should be used under cardiac monitoring in the management of acute severe hypocalcemia, defined as the presence of significant neurologic, cardiac or respiratory symptoms, or serum calcium < 1.75 mmol/L [14, 33, 39].

#### Thiazide diuretics

**Fig. 2:**
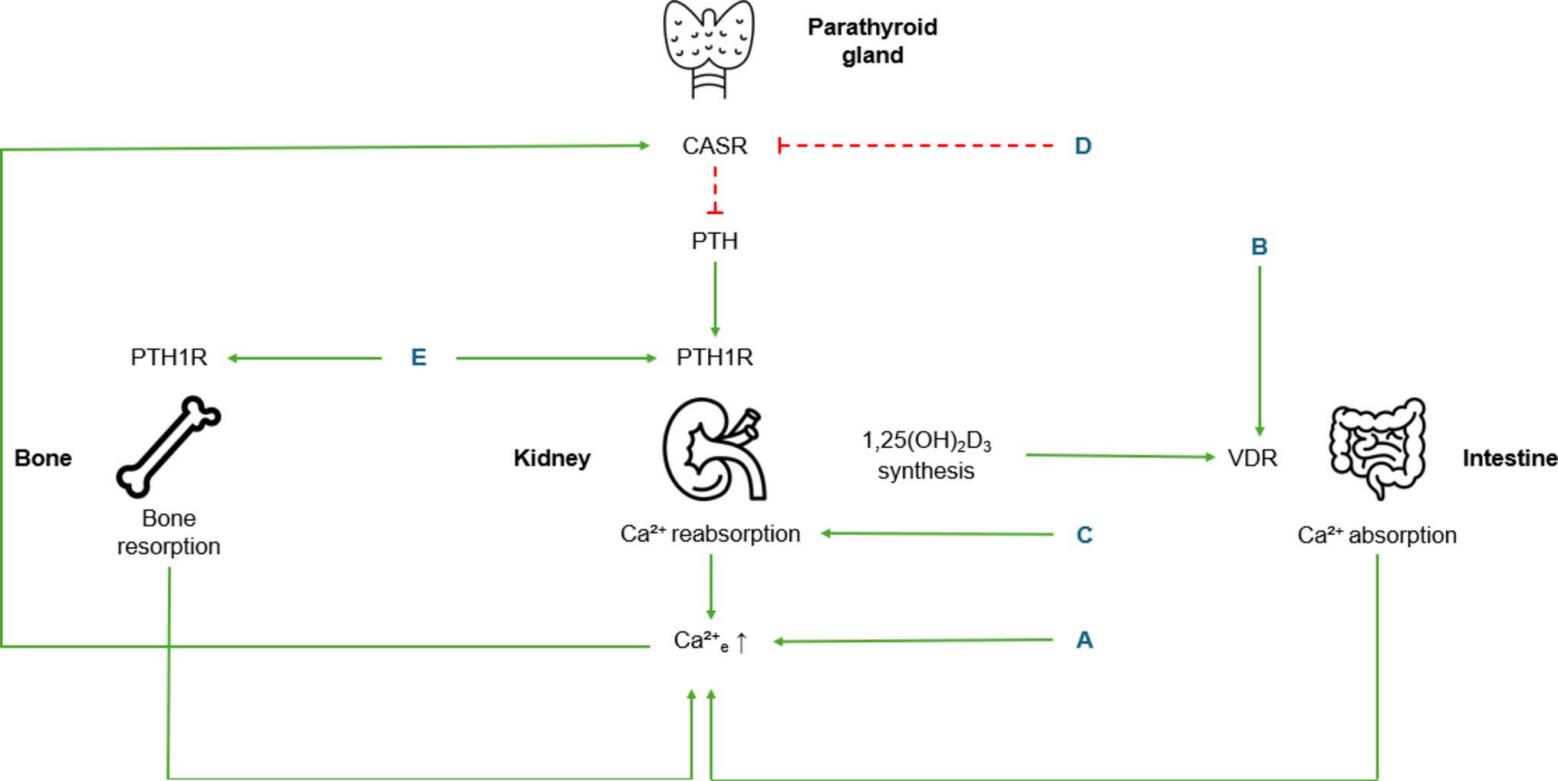
Treatment options for Autosomal Dominant Hypocalcemia type 1. A: calcium supplements; B: active vitamin D; C: thiazide diuretics; D: calcilytics; E: PTH analogues. Solid arrows indicate stimulation, dashed lines indicate inhibition. CASR: calcium sensing receptor; PTH1R: PTH1 receptor; VDR: vitamin D receptor Images: Flaticon.com

Thiazide diuretics reduce urinary calcium excretion by increasing the renal calcium reabsorption (Fig. 2). According to Roszko et al. 21% of ADH1 patients received thiazide diuretics in combination with active vitamin D and calcium supplements [3]. Thiazides can induce hypokalemia and increase urinary magnesium loss [40, 41]. Therefore, ADH1 patients with Bartter-like syndrome or with high urinary magnesium excretion and hypomagnesaemia are advised to avoid thiazide diuretics [18, 33]. It is advised to combine thiazide diuretics with a low-sodium diet since thiazides cause increased sodium clearance [42].

#### Phosphorus binders

There are no studies regarding the use of phosphorus binder drugs such as lanthanum carbonate and sevalemer hydrochloride in ADH1. Even in the broader field of hypoPT, research on phosphorus binders is limited. Sakane et al.. propose the use of phosphorus binders in cases of refractory hyperphosphatemia [35]. However, Gafni et al. suggest that calcium supplementation may be a better approach for the management of hyperphosphatemia since calcium is an effective phosphorus binder [43].

#### Dietary recommendations

Sodium transporters in the proximal renal tubule reabsorb calcium from the glomerular filtrate together with sodium independently of PTH. As such, a low-sodium diet reduces natriuresis and calciuresis [42]. Therefore, ESE, the First International Conference and the Second International Workshop guidelines recommend a low-sodium diet in hypoPT patients with hypercalciuria [14, 33, 34]. In hypoPT patients with hyperphosphatemia and/or an elevated calcium-phosphate product, the guidelines recommend a low phosphorus diet, avoiding eggs, meat and phosphorus-rich soda drinks [33–35]. Given dairy products are rich in both calcium and phosphorus, increasing dietary calcium may lead to hyperphosphatemia [38]. Therefore, in hypoPT patients, oral calcium supplements are preferred since they bind dietary phosphorus, reducing intestinal phosphorus absorption [35].

Conventional treatment is able to increase the serum calcium levels but does not correct the underlying pathophysiology of ADH1. It creates a perceived hypercalcemia that further lowers PTH levels and exacerbates hypercalciuria, increasing the risk for nephrocalcinosis, nephrolithiasis and reduced renal function in the long term.

### Emerging treatments for ADH1

#### Parathyroid hormone analogues

##### PTH1-34

1-34, also known as teriparatide, is the biologically active N-terminal fragment of the PTH peptide [19]. Winer et al. compared once-daily subcutaneous (SC) PTH1-34 to twice-daily oral calcitriol combined with supplemental calcium carbonate in a randomized cross-over trial [44]. This study included 10 hypoPT patients and showed that PTH1-34 maintained serum calcium within the normal range with decreased urinary calcium excretion compared to calcitriol. In another study in 17 hypoPT patients of whom 5 had ADH1, Winer et al. showed that a twice-daily PTH1-34 regimen led to a more stable control of serum calcium levels over a 24-hour period compared to a once-daily regimen [45]. This trial also showed that patients with ADH1 benefited most from twice- compared to once-daily injection, and that the rise in serum calcium levels was not accompanied by an increased urinary calcium excretion. A similar study was conducted in 14 children with hypoPT of whom one had ADH1 [46]. This study concluded that a twice-daily PTH1-34 regimen leads to a greater reduction in variation of serum calcium levels. In both studies, serum phosphorus levels remained elevated and the need for magnesium supplementation seemed to increase under PTH1-34. Furthermore, Winer et al. compared twice-daily SC PTH1-34 with continuous SC pump delivery in 12 children and young adults with severe congenital hypoPT of whom 7 suffered from ADH1 [47]. Pump delivery resulted in near normalization of mean serum calcium, normalized the mean 24-hour urine calcium, reduced urinary magnesium excretion, and normalized serum magnesium levels. However, serum phosphorus levels increased while urinary phosphate excretion was similar and remained within the normal range for both delivery methods. In ADH1 patients, there was no difference in urinary calcium excretion to serum calcium ratio between the delivery methods. In a retrospective cohort study in 6 ADH1 patients with seizures Sastre et al. assessed the effectiveness of PTH1-34 pump delivery compared to calcium and vitamin D analogue treatment in the same patients [48]. They showed that SC PTH1-34 infusion increased serum calcium levels and decreased serum phosphate and calcium-phosphate product. Furthermore, SC PTH1-34 infusion reduced the number of seizures from 2 to 0.01 per month, resulting in discontinuation of anticonvulsant therapy and fewer emergency hospital admissions. Moreover, SC PTH1-34 infusion neither worsened nephrocalcinosis nor increased urinary calcium excretion.

##### Recombinant human PTH1-84

Recombinant human PTH1-84 (rhPTH1-84), the full-length 84 amino-acid PTH peptide, has a longer half-life than PTH1-34 [18]. Mannstadt et al. tested the efficacy, safety and tolerability of once-daily SC 50–100 µg rhPTH1-84 in 134 hypoPT patients [49]. However, ADH1 patients were excluded. This trial showed that association of rhPTH1-84 to calcium supplements and vitamin D results in optimalization and stabilization of serum calcium levels while substantially reducing the need for calcium and vitamin D supplementation. Moreover, rhPTH1-84 resulted in increased serum calcium levels, decreased serum phosphate levels, decreased calcium-phosphate product and activation of endogenous 1,25(OH)_2_D_3_ production. Hawkes et al. reported on 3 ADH1 patients treated with once-daily SC rhPTH1-84, resulting in increased serum calcium, decreased calciuria, and discontinuation or reduction of need for calcium supplements and calcitriol [50].

##### TransCon PTH

TransCon PTH is an inactive prodrug consisting of a parent drug (PTH1-34) transiently bound to an inert carrier (polyethylene glycol(PEG)). This carrier hinders PTH1-34 binding to the PTH1 receptor (PTH1R), renal clearance and enzymatic degradation. Thereby, the carrier prolongs the half-life of PTH1-34 to 60 h leading to stable serum PTH levels within the normal physiological range [51]. In a phase 3 clinical trial, Khan et al. investigated the efficacy and safety of SC TransCon PTH in 84 hypoPT patients of whom one had ADH1 [52]. They showed that at 26 weeks TransCon PTH maintained normocalcemia without active vitamin D and ≤ 600 mg/d of calcium supplementation. Moreover, serum phosphate, calcium-phosphate product, magnesium, 25(OH)D_3_ and 1,25(OH)_2_D_3_ levels remained within the normal range. Also, 60.7% of participants receiving TransCon PTH achieved normal 24-hour urine calcium excretion. Furthermore, TransCon PTH resulted in a significant improvement in physical functioning and well-being.

##### Other PTH analogues

A phase 1 clinical trial showed that a single SC injection of AZP-3601, a 36-amino acid PTH analogue, led to a dose-dependent sustained increase of serum calcium levels for at least 24 h [53]. Shimizu et al. found that daily SC Ala^1,3,12,18,22^Gln^10^Arg^11^Trp^14^Lys^26^]-PTH(1–14)/PTHrP (15–36), a long-acting form of PTH (LA-PTH), increased serum calcium levels in thyroparathyroidectomized (TPTX) rats [54]. Krishnan et al. showed in TPTX rats that once-daily SC LY627-2 K, another LA-PTH, resulted in a greater increase in serum calcium without increasing urinary calcium excretion compared to equimolar dosages of PTH1-84 [55]. PCO371, an oral PTHR1-agonist, increased serum calcium levels without increasing urinary calcium excretion, and decreased serum phosphate in TPTX rats [56]. A recent clinical trial with PCO371 was terminated because of an uncertain risk-benefit balance, not further specified [57]. Finally, Ish-Shalom et al. showed in 15 hypoPT patients that hPTH1-34 acetate, an oral PTH1-34 formulation, as adjunct to conventional treatment, leads to a 42% reduction in calcium supplementation while median serum calcium levels remained above the lower target (> 1.875 mmol/L) [58]. Serum phosphate levels decreased 23% two hours after the first dose and remained within normal limits throughout the remainder of the sixteen-week study period. 24-hour urinary calcium excretion tended to decrease as well (-21%).

An overview of clinical studies with PTH analogues in ADH1 patients is provided in Table 2.

**Table 2:** Studies with parathyroid hormone analogues in autosomal Dominant Hypocalcemia type 1 patients

First author Year [Ref]	Number of patients	Number of ADH1 patients	Study design	Study duration	Treatment	Result
Winer 1996 [44]	10	0-3^a^	Randomized cross-over trial	20 weeks	Once-daily SC PTH1-34 versus twice-daily PO calcitriol combined with supplemental calcium carbonate	= Serum calcium ↓ Urinary calcium excretion
Winer 1998 [45]	17	5	Randomized cross-over trial	28 weeks	Twice-daily SC PTH1-34 regimen versus once-daily	More stable control of serum calcium levels over a 24-hour period
Winer 2008 [46]	14	1	Randomized cross-over trial	28 weeks	Twice-daily PTH1-34 regimen versus once-daily	Greater reduction in variation of serum calcium levels
Winer 2014 [47]	12	7	Randomized cross-over trial	26 weeks	Pump delivery of SC PTH1-34 versus twice-daily regimen	Normal mean serum calcium levels Normal mean 24-hour urine calcium ↓ Urinary magnesium excretion Normal serum magnesium levels ↑ Serum phosphorus levels Normal urinary phosphate excretion for both delivery methods In ADH1 patients: no difference in urinary calcium excretion to serum calcium ratio between the delivery methods
Sastre 2021 [48]	6	6	Retrospective cohort study	0.8–5.5 years	SC PTH1-34 pump delivery versus PO calcium and vitamin D analogue therapy	↑ Serum calcium levels ↓ Serum phosphate and calcium-phosphate product ↓ Number of seizures from 2.0 to 0.01 per month, resulting in discontinuation of anticonvulsant therapy and fewer emergency hospital admissions No worsening of nephrocalcinosis or increased urinary calcium excretion
Hawkes 2020 [50]	3	3	Retrospective case series	10–18 months	Once-daily SC PTH1-84	= or ↑ Serum calcium levels ↓ Urinary calcium excretion Discontinuation or ↓ dose of calcium supplements and calcitriol
Khan 2022 [52]	84	1	Phase 3 clinical trial	26 weeks	Once daily SC TransCon PTH at individually titrated dosages with a median of 21 µg/d	Maintenance of normocalcemia without active vitamin D and ≤ 600 mg/d of calcium supplementation Normal serum phosphate, calcium-phosphate product, magnesium, 25(OH)D_3_ and 1,25(OH)_2_D_3_ levels Normal 24-hour urine calcium excretion in 60.7% ↑ physical functioning and well-being

^a^ Three patients reported as ‘familial cause of hypoPT’ (ADH1 not specifically mentioned)

Abbreviations: SC: subcutaneous; PO: peroral; PTH: parathyroid hormone

#### Calcilytics

Calcilytics act as negative allosteric modulators of the CASR (Fig. 2) [59]. By inhibiting CASR activity, calcilytics cause a rightward shift in the Ca^2+^ _e_-concentration-response curve, thus correcting the molecular mechanism of *CASR* gain-of-function variants [60, 61].

##### Preclinical data

Dong et al. were the first to report the efficacy of calcilytics in CASR knock-in mouse models with ADH1 phenotype, showing that both SC administration of 50 µg/kg PTH1-34 and oral administration of 20 µg/g of the calcilytic JTT-305/MK-5442 increased serum calcium, magnesium and 1,25(OH)_2_D_3_ levels and reduced serum phosphate levels [62]. In addition, they found that JTT-305/MK-5442 significantly diminished urinary calcium excretion while PTH1-34 did not. As a result, knock-in mice treated with PTH1-34 developed renal calcifications, whereas knock-in mice treated with JTT-305/MK-5442 did not. Hannan et al. studied the effect of intraperitoneal injection (IP) of 30 mg/kg of the calcilytic NPS 2143 in WT, Nuf/+ and Nuf/Nuf mice harboring the L723Q *CASR* gain-of-function variant [63]. They found that IP NPS 2143 led to a significant rise in serum PTH and calcium levels at 1 h after injection, which remained at 4 h and returned to baseline 24 h after injection. However, there was no effect on urinary calcium excretion and serum phosphate levels even increased. Hannan et al. showed that SC bolus of 25 mg/kg the calcilytic NPSP795 also increases serum calcium levels in WT, Nuf/+ and Nuf/Nuf mice [12].

##### Clinical trials 

Roberts et al. investigated pharmacokinetics, pharmacodynamics, efficacy and safety of NPSP795 in 5 adults with 4 different CASR gain-of-function variants [64]. They showed that IV infusion of 5-30 mg NPSP795 led to dose-dependent increases in serum PTH levels. Nevertheless, PTH levels decreased rapidly after ceasing the infusion and a pronounced variability in response was observed, not explained by differences in drug levels. This variability was especially noteworthy in 2 subjects with the same CASR pathogenic variant (A840V). IV NPSP75 led to stable serum calcium levels despite cessation of calcitriol and only low dose calcium supplementation. However, neither a significant increase in serum calcium nor a decrease in fractional excretion of calcium was observed.

A study regarding safety, tolerability and efficacy of calcilytic CLTX-305 (Encaleret) in 13 patients with ADH1 showed that Encaleret led to normocalcemia and reduced hypercalciuria [65]. In addition, Encaleret increased serum PTH, magnesium and 1,25(OH)_2_D_3_ levels while tubular phosphate reabsorption and phosphate serum levels decreased.

An overview of clinical studies with calcilytics in ADH1 patients is provided in Table 3.

**Table 3:** Studies with calcilytics in autosomal Dominant Hypocalcemia type 1 patients

First author Year [Ref]	Number of ADH1 patients	Study design	Study duration	Treatment	Result
Roberts 2019 [64]	5	Open-label, nonrandomized, single‐center, intrasubject dose‐escalating phase 2b study	5 days	IV NPSP795	Dose-dependent increases in serum PTH levels Stable serum calcium levels despite cessation of calcitriol and limited calcium supplementation No significant ↑ serum calcium No significant ↓ fractional calcium excretion
Gafni 2023 [65]	13	Open-label, Phase 2b study	Two 5-day inpatient dose-ranging periods + 24 week outpatient period	PO Encaleret	Normalization of serum calcium ↓ Hypercalciuria ↑ Serum magnesium, intact PTH, 1,25(OH)_2_D_3_ levels ↓ Serum phosphate levels ↓ Tubular phosphate reabsorption

Abbreviations: IV: intravenous; PO: peroral

### Monitoring ADH1 patients

Currently, no studies providing guidance for monitoring in ADH1 patients have been published. Even in the broader context of patients with chronic hypoPT, studies on monitoring strategies for preventing complications are limited. Recently, Van Uum et al. conducted a systematic current practice survey (SCPS) among 97 international hypoPT experts to assess the current clinical practice regarding monitoring of hypoPT patients [66]. Results of this SCPS led to the recommendation to measure serum calcium (ionized or albumin-corrected), creatinine, magnesium, phosphate, 25(OH)D_3_, and 24-hour urine calcium and creatinine at the initial assessment. Regarding follow-up, they recommended to measure serum calcium (ionized or albumin-corrected), creatinine, magnesium and phosphate every 3 to 12 months, and 25(OH)D_3_ every 6 to 12 months. They also recommended 24-hour urinalysis of calcium and creatinine every 6 months to 2 years. In nonstable patients, serum calcium and phosphate measurements should be assessed as often as clinically indicated. Besides these recommendations based on the SCPS, the research panel formulated two additional recommendations. Firstly, they advise screening for nephrocalcinosis and nephrolithiasis by renal ultrasonography (RUS) or CT at the initial assessment. Secondly, serum calcium should be remeasured within days after adjustments in medical treatment. These recommendations are largely consistent with previous guidelines [14, 33, 34]. ESE recommended the same biochemical measurements but at a 3 to 6 months interval, and additional biochemical monitoring weekly to every other week after alterations in therapy [34]. Moreover, renal imaging is advised when patients experience symptoms of nephrolithiasis or when serum creatinine levels rise. Although time intervals were not specified, ESE recommended more frequent monitoring of patients with ADH1 treated with calcium and/or active vitamin D analogues since they are at higher risk of hypercalciuria and renal complications. The 2016 guideline of the First International Conference on the Management of HypoPT additionally recommended monitoring of basal ganglia calcification with central nervous system imaging and of cataracts with an ophthalmological exam, depending on findings at initial assessment and the clinical presentation [33]. The Second International Workshop also suggested a slit-lamp examination in search of ocular complications in patients with visual symptoms [14].

## Disscusion

Conventional treatment of symptomatic ADH1 consisting of vitamin D analogues and calcium supplementation is largely based on guidelines regarding management of chronic hypoPT (of which the major part is post-surgical hypoPT in adult patients) such as the ESE guidelines, the First International Conference on the Management of Hypoparathyroidism and the Second International Workshop [14, 33, 34]. The ESE guidelines state that ADH1 patients have a higher risk of renal calcifications on conventional treatment and that thiazides can be used to lower the urinary calcium excretion. These recommendations are focused primarily on the symptomatic ADH1 patient. However, there are no recommendations for asymptomatic ADH1 patients and the question remains whether and how we should treat these patients. In addition, unlike the majority of hypoPT patients, ADH1 is a congenital disorder with a longer disease duration and therefore a higher risk of complications.

Studies with PTH1-34 and rhPTH1-84 in hypoPT patients showed that both can maintain normal serum calcium levels without increasing urinary calcium excretion [44–49]. However, these studies included few ADH1 patients and analyses in ADH1 subgroups were rarely performed. The randomized controlled trial with rhPTH1-84 even specifically excluded ADH1 patients [49]. However, a small case series with 3 ADH1 patients treated with rhPTH1-84 showed promising results [50]. A retrospective cohort study in 6 ADH1 patients found that PTH1-34 infusion increased serum calcium levels and led to fewer seizures compared to conventional treatment in the same patients [48]. Taken together, PTH1-34 might have a place in the prevention of seizures in ADH1 patients but there is currently insufficient evidence to recommend PTH1-34 as a first-line treatment in ADH1 patients. Moreover, as PTH1-34 has only market authorization for short term use in osteoporosis, only off-label use is possible. Besides, rhPTH1-84 will be withdrawn from the global market by the end of 2024 due to unresolved manufacturing challenges.

TransCon PTH was shown to maintain normal serum calcium levels, normalize 24-hour urine calcium excretion and reduce the need for conventional treatment resulting in improvement of physical functioning and well-being in 84 hypoPT patients of whom only one had ADH1 [52]. TransCon PTH, granted FDA and EMA approval for treatment of hypoPT in December 2023, represents a promising drug to offer to highly symptomatic ADH1 patients. However, longer-term studies with more ADH1 patients are needed, which should include end-organ effects such as on kidney, brain and bone.

Oral hPTH1-34 acetate was shown to be effective in reducing calcium supplementation while maintaining normal serum calcium levels, but the study did not report on subjects with ADH1 and did not reach a statistically significant decrease in urinary calcium excretion [58]. Other PTH analogues have only been studied in animal studies [53–55].

Since calcilytics correct the molecular mechanism of *CASR* gain-of-function variants they represent a treatment option with great potential for ADH1 patients. Preclinical studies in ADH1 rodent models indeed showed promising results with elevation of serum calcium, magnesium and 1,25(OH)_2_D_3_ levels, and reduction of serum phosphate and urinary calcium excretion [62]. However, the first published clinical study on calcilytics in ADH1 patients showed no significant increase in serum calcium levels nor reduction in urinary calcium excretion compared to conventional treatment [64]. Nonetheless this study was performed in only 5 ADH1 patients. A trial with Encaleret in 13 ADH1 patients showed normalization of serum calcium levels and reduction of hypercalciuria, tubular phosphate reabsorption and serum phosphate levels [65]. In addition, Encaleret led to an increase in serum PTH, magnesium and 1,25(OH)_2_D_3_ levels. Altogether, there is currently potentially promising evidence from clinical studies for calcilytics in ADH1. Randomized controlled trials with more participants and longer follow-up remain needed.

### Practical recommendations

Based on the results from clinical studies in ADH1 patients, and guidelines and recommendations for chronic hypoPT patients, we propose practical recommendations for treatment and follow-up of ADH1 patients.

**Fig. 3:**
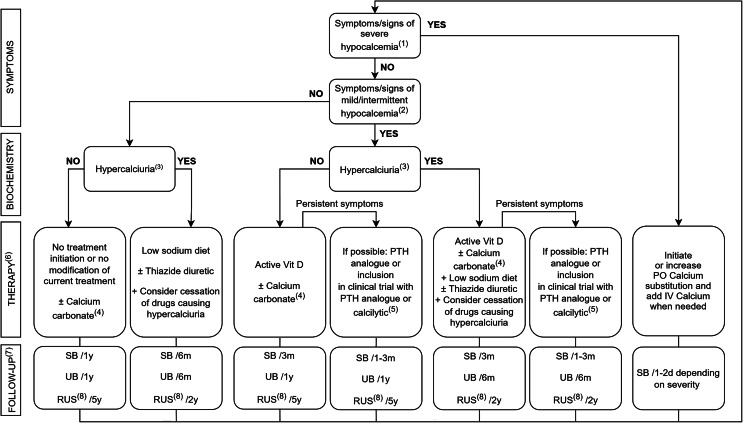
Management of Autosomal Dominant Hypocalcemia type 1.^(1)^ Severe hypocalcemia is defined as the presence of significant neurologic, cardiac or respiratory symptoms or serum calcium < 1.75mmol/L ^(2)^ Mild neuromuscular symptoms, central nervous system calcifications, nephrocalcinosis, kidney stones, impaired renal function, posterior subcapsular cataract ^(3)^ Hypercalciuria is defined as > 6.25mmol/24 h urine for females, > 7.5mmol/24 h urine for males (or a urinary calcium/creatinine ratio > 0.6 when a reliable urine collection is not possible) ^(4)^ Add calcium carbonate if daily calcium dietary intake is < 1000 mg or if hyperphosphatemia is present despite dietary recommendations ^(5)^ Follow-up as determined in clinical trial protocol ^(6)^ Therapy directed towards target: symptom relief and serum [Ca^2+^] between 1.8–2.1 mmol/L. We advise a 25(OH)D_3_ concentration between 20–50 ng/mL using ergocalciferol or cholecalciferol if required ^(7)^ Follow-up includes a brain CT and slit lamp examination every 10 years independently of signs and symptoms of hypocalcemia or presence of hypercalciuria ^(8)^ Renal ultrasonography should be repeated when patients experience symptoms of nephrolithiasis or when renal function declines, regardless of time intervals. IV: intravenous; m: month; RUS: renal ultrasonography; SB: serum biochemistry, including calcium, albumin, phosphate, magnesium, potassium, sodium, PTH, creatinine, 25(OH)D_3_; UB: urine biochemistry including calcium, sodium and creatinine; y: year

We recommend a baseline measurement of serum calcium, albumin, phosphate, magnesium, potassium, sodium, PTH, creatinine, 25(OH)D_3_, and 24-hour urinary calcium, sodium, and creatinine. Further follow-up depends on symptoms, signs and biochemical characteristics, as described in Fig. 3. We recommend screening for complications of ADH1 at diagnosis with RUS, brain CT and ophthalmologic examination. RUS should be repeated every 5 years, or every 2 years in case of hypercalciuria (defined as > 6.25mmol/24 h urine for females, > 7.5mmol/24 h urine for males, or a urinary calcium/creatinine ratio > 0.6 when a reliable urine collection is not possible) [6, 14]. RUS should be repeated regardless of time intervals when patients experience symptoms of nephrolithiasis or when renal function declines. We recommend repeating brain CT and ophthalmological investigation every 10 years, or additionally in case of signs or symptoms.

In asymptomatic patients without hypercalciuria we recommend not to commence medical treatment if serum calcium is within the target range (1.8–2.1 mmol/L). When the serum calcium is < 1.8mmol/L calcium supplements should be started. In case of asymptomatic patients with hypercalciuria and intake of drugs that increase urinary calcium and sodium levels such as loop diuretics, glucocorticoids, carbonic anhydrase inhibitors and sodium-glucose co-transporter-2 (SGLT2) inhibitors, we recommend cessation of these drugs if possible. If not possible or if hypercalciuria persists after cessation of these drugs, starting a thiazide diuretic is recommended. The use of a thiazide diuretic should be combined with a low-sodium diet, except in case of low blood pressure. Serum potassium and magnesium levels should be monitored within 5–10 days after starting treatment. When decreased, potassium and magnesium supplementation should be associated. If a thiazide diuretic and low-sodium diet prove insufficient and serum calcium is < 1.8 mmol/L we advise starting active vitamin D targeting serum calcium levels between 1.8 and 2.1 mmol/L. We recommend against the use of thiazides in ADH1 patients with Bartter-like syndrome with hypokalemia and ADH1 patients with pre-existing hypomagnesaemia and/or high urinary magnesium excretion.

In symptomatic patients we advise starting with active vitamin D analogues (calcitriol twice-daily or alphacalcidol once-daily) and titration according to the serum calcium level, targeting a serum calcium level between 1.8 and 2.1 mmol/L and alleviating symptoms. Calcium supplements should be associated if the estimated daily calcium dietary intake is below 1000 mg or if hyperphosphatemia is present. As for calcium supplementation, we recommend calcium carbonate as first choice. Calcium citrate can be an alternative in patients with achlorhydria, treated with PPIs or complaining of constipation under calcium carbonate. Since urinary calcium excretion in ADH1 is often increased, we suggest 1000 mg of elemental calcium instead of 800–2000 mg as stated in the ESE guidelines. If hypercalciuria is present or symptoms are not yet controlled, we recommend combination with a thiazide diuretic. In symptomatic patients who are not controlled with active vitamin D, calcium supplements and a thiazide diuretic, we recommend adding SC PTH1-34 twice-daily, particularly in case of seizures. Inclusion in a clinical trial with calcilytics, if possible, is an alternative when PTH1-34 is not available. In patients with acute hypocalcemia defined as the presence of significant neurologic, cardiac or respiratory symptoms or serum calcium < 1.75mmol/L, IV calcium gluconate should be administered under cardiac monitoring. A 10% calcium gluconate solution in 50 mL of 5% dextrose over 10–20 min followed by calcium gluconate infusion at 0.5–1.5 mg/kg/h over 8–10 h is recommended [14, 33].

We advise a 25(OH)D_3_ target concentration of 20 ng/mL as stated in the ESE guidelines, using ergocalciferol or cholecalciferol if required. In case of hyperphosphatemia, we recommend a low phosphorus diet and calcium carbonate to reduce intestinal phosphorus absorption. We do not recommend the use of phosphorus binders other than calcium carbonate.

### Limitations

The literature search was done by one researcher (TDC). Practice recommendations are based on case reports and clinical studies including only a small number of ADH1 patients. Preferential reporting of more severely symptomatic cases over asymptomatic cases may have induced a bias with overestimation of the need for treatment. Our practice recommendations do not include specific clinical contexts and subgroups such as pregnancy, the ICU setting, concurrent treatment with other drugs such as bone antiresorptive drugs and lithium or advanced renal or intestinal insufficiency (e.g. end-stage chronic kidney disease, bariatric surgery).

## Conclusion

We provided an overview of current conventional treatment options of ADH1 and discussed the currently available evidence regarding new treatments with PTH analogues and calcilytics. Based on these data, we proposed practical recommendations to assist clinicians in the management of ADH1 patients, a patient group with a different pathophysiology, longer disease duration and different treatment targets as compared to the ‘classical’ hypoPT patient. Due to the rarity of ADH1, the small number of studies, and the emerging treatment modalities, there is a need to include ADH1 patients in international registries, observational and interventional clinical trials.

## Electronic supplementary material

Below is the link to the electronic supplementary material.

Supplementary Material 1
