# Supplementary material for: Management of autosomal dominant hypocalcemia type 1: Literature review and clinical practice recommendations
Source: J Endocrinol Invest. 2024 Nov 28;48(4):831–44. doi: 10.1007/s40618-024-02496-y (PMC11950097; doi:10.1007/s40618-024-02496-y)
Supplement: Supplementary file 1 — Supplementary Material 1 [file 40618_2024_2496_MOESM1_ESM.docx]

**SUPPLEMENTAL DATA**

# Search strategy

In Pubmed, we used the following search terms: "Hypercalciuric Hypocalcemia, Familial" [Supplementary Concept]; "Hypercalciuric Hypocalcemia, Familial" [Supplementary Concept]) AND "therapy" [Subheading]; “autosomal dominant hypocalcemia type 1”; "autosomal dominant hypocalcaemia type 1"; "autosomal dominant hypocalcaemia type 1" AND "treatment"; "autosomal dominant hypocalcemia type 1" AND "treatment"; "autosomal dominant hypocalcemia" AND "management"; "autosomal dominant hypocalcaemia type 1" AND "management"; "autosomal dominant hypocalcemia" AND "parathyroid hormone"; "autosomal dominant hypocalcemia type 1" AND "parathyroid hormone"; "autosomal dominant hypocalcaemia type 1" AND "parathyroid hormone"; "autosomal dominant hypocalcemia" AND "PTH"; "autosomal dominant hypocalcemia type 1" AND "PTH"; "autosomal dominant hypocalcaemia" AND "PTH"; "autosomal dominant hypocalcaemia type 1" AND "PTH"; "autosomal dominant hypocalcemia" AND "calcilytics”; "autosomal dominant hypocalcemia type 1" AND "calcilytics”; "autosomal dominant hypocalcaemia" AND "calcilytics”; "autosomal dominant hypocalcaemia type 1" AND "calcilytics”; "autosomal dominant hypocalcemia" AND "vitamin D”; "autosomal dominant hypocalcemia type 1" AND " vitamin D”; "autosomal dominant hypocalcaemia" AND " vitamin D”; "autosomal dominant hypocalcaemia type 1" AND " vitamin D”; "autosomal dominant hypocalcemia" AND " calcium”; "autosomal dominant hypocalcemia type 1" AND "calcium”; "autosomal dominant hypocalcaemia" AND "calcium”; "autosomal dominant hypocalcaemia type 1" AND "calcium”; "autosomal dominant hypocalcemia" AND "calcium” AND “vitamin D”; “autosomal dominant hypocalcemia type 1" AND "calcium” OR “vitamin D”; "autosomal dominant hypocalcaemia" AND "calcium” AND “vitamin D”; "autosomal dominant hypocalcaemia type 1" AND "calcium” AND “vitamin D”; "autosomal dominant hypocalcemia" AND "diuretic"; "autosomal dominant hypocalcemia type 1" AND "diuretic"; "autosomal dominant hypocalcaemia" AND "diuretic"; "autosomal dominant hypocalcaemia type 1" AND "diuretic"; "autosomal dominant hypocalcemia" AND "thiazide”; "autosomal dominant hypocalcemia type 1" AND " thiazide”; "autosomal dominant hypocalcaemia" AND "thiazide”; "autosomal dominant hypocalcaemia type 1" AND " thiazide”; "Hypoparathyroidism familial isolated" [Supplementary Concept]; "Hypoparathyroidism familial isolated" [Supplementary Concept] AND "management or treatment"; "Hypoparathyroidism"[Mesh] AND ("management" OR "treatment").

In Embase we used the following search terms: 'autosomal dominant hypocalcemia' AND 'parathyroid hormone[1-34]'/exp; 'autosomal dominant hypocalcemia type 1'/exp AND 'parathyroid hormone[1-34]'/exp; 'autosomal dominant hypocalcemia type 1' AND 'vitamin d'/exp AND 'calcium'/exp; 'autosomal dominant hypocalcemia type 1'/exp AND ('vitamin d'/exp OR 'calcium'/exp); 'autosomal dominant hypocalcemia type 1'/exp AND 'calcilytic agent'/exp; 'autosomal dominant hypocalcemia'/exp AND 'calcilytic agent'/exp; 'autosomal dominant hypocalcemia type 1'/exp AND 'thiazide diuretic agent'/exp.

In Cochrane Library we used the following search terms: “autosomal dominant hypocalcemia”; MeSH descriptor: “hypocalcemia” /// qualifier: “therapy”; ("CASR protein, human" [Supplementary Concept]) AND "Hypocalcemia"[Mesh].

In Trip database we used the following search terms: ‘autosomal dominant hypocalcemia’; ‘autosomal dominant hypocalcemia type 1’; ‘autosomal dominant hypocalcemia type 1, genetics’; ‘autosomal dominant hypocalcemia type 1, management’.

In controlled-trials.com, clinicaltrials.gov, PROSPERO, MedRxiv and BioRxviv we used the search term “autosomal dominant hypocalcemia”.
